# Reliability of ultrasonography measurement of the anterior talofibular ligament (ATFL) length in healthy subjects (in vivo), based on examiner experience and patient positioning

**DOI:** 10.1186/s40634-019-0199-z

**Published:** 2019-07-02

**Authors:** Karl-Heinz Kristen, Jesse Seilern und Aspang, Johannes Wiedemann, Florian Hartenbach, Hannes Platzgummer

**Affiliations:** 1 Sportklinik Wien, Werdertorgasse 14/8, A-1010 Vienna, Austria; 2 Department of Musculoskeletal Radiology, Medical University of Vienna, Spitalgasse 23, 1090 Vienna, Austria

**Keywords:** Ultrasonography, Lateral ligament, ankle, Patient positioning, Reproducibility of results, Observer variation

## Abstract

**Background:**

The most common cause of ankle injury is the supination trauma, inflicting a partial or complete rupture of the anterior talofibular ligament (ATFL). Among conventional diagnostic tools and procedures of sports injuries, the method of stress-ultrasonography is reportedly a promising diagnostic tool for examining injuries of the lateral ligaments of the ankle. Preceding studies predominantly examined the comparability of stress-ultrasonography and other established diagnostic tools in terms of efficacy, viability and quality. The purpose of this study was to assess the reliability of stress-ultrasonography of the ATFL based on varying examiner experience and patient positioning.

**Method:**

Sixteen healthy subjects were examined by four examiners with differing levels of skill and experience in ultrasonography, ranging from laymen to specialist. Measurements were recorded and interrater correlation coefficient (ICC) was applied in four positions, including a neutral position (A), medial rotation (B), plantar flexion (C) and inversion of the foot (D).

**Results:**

The length of the ATFL was 14.958 ± 2.145 mm in position A, 15.886 ± 1.994 mm in position B, 16.270 ± 1.858 mm in position C and 15.170 ± 1.781 mm in position D. The average length change was 0.928 ± 0.804 mm (6.656 ± 6.299%) in position B, 1.313 ± 1.266 mm (9.746 ± 9.484%) in position C and 0.213 ± 1.807 mm (2.604 ± 12.308%) in position D. The correlation of the combined results of all four investigators was 0.333 for position A, 0.386 for position B, 0.320 for position C and 0.517 for position D. The highest ICC (0.811) was recorded between the orthopedic specialist and the radiology specialist. The lowest ICC (0.299) was recorded between the laymen and the radiology specialist.

**Conclusion:**

The reliability of the ATFL examination seems to be exceedingly dependent on the examiner’s experience and skill in ultrasonographic (US) diagnostic. Moreover, the inversion positioning of the foot, described by the European Society of Musculoskeletal Radiology (ESSR) yielded the highest measurement reliability.

## Background

Injuries to the ligamentous structures of the ankle joint belong to the most prevalent injuries in sports, with the anterior talofibular ligament (ATFL) being afflicted in 65% of the cases in consequence of supination trauma (Roos et al., 2017). Caused by inadequate injury management as well as repeated injury, 10–20% of ankle injuries lead to chronic instability of the joint (Walther et al., 2013). In order to prevent long-term complications of injury to the ATFL, such as chronic instability and osteoarthritis, therapeutic planning is vital and ought to commence swiftly and precise (Kerkhoffs et al., 2002; Delahunt et al., 2018).

Standard diagnostic methods of lateral ligament injury of the ankle described in the literature include clinical examination, X-ray, magnetic resonance imaging (MRI) and arthrometer stress testing. However, refutations to aforementioned diagnostic tools include mediocre reproducibility, financial- and time expenditure, and in the case of X-ray, health exposure (Kerkhoffs et al., 2002). Previously indicated by various authors, ultrasonographic (US) stress-test of the ligament apparatus of the talocrural joint is a viable supplement to other imaging diagnostics and clinical examination, due to the cost efficient, noninvasive and timesaving nature (Friedrich et al., 1990). Furthermore, preliminary findings underline the accuracy of US stress-testing, compared to above-mentioned diagnostic tools (Cheng et al., 2014). The available results on this topic stem from different levels of professional examiners with the majority comprised of professional sonographers.

As of current, no statement has been made, as to whether, and to what extent examiner experience effects the reliability of US ATFL-examination. To take advantage of the effectiveness of this diagnostic tool and to administer adequate therapeutic management, evidence-based guidance in forecasting reliability based on the examining physician’s skill, would be advantageous. Therefore, we intend to assess the reliability of stress-ultrasonography measurement of the ATFL, based on varying examiner experience and skill levels in four patient positions, using the model of interrater reliability. We hypothesize that increasing degree of experience and skill is positively correlated with good/excellent interrater reliability.

## Methods

### Patient selection

Between November 2017 and February 2018, a total of 16 subjects were selected for US examination of ATFL, consisting of 11 males and 5 females with the median age of 25 years, ranging from 19 to 55 years. Each pupil was required to complete a written questionnaire, including age, height, athletic activity and injury history involving acute injury or chronic instability of the ankle. Pupils without acute injury or prior injury history of at least one of their ankles, were included into the study. In the case of acute injury or chronic instability of one and the same ankle in the pupil’s history, the healthy ankle was examined. If the subject reported no history of ankle injuries or chronic instability in either ankle, the joint to be examined was selected at random.

### Investigators

The US evaluation of the ATFL of each subject was conducted by four respective male investigators with distinct skill and experience in US diagnostics (Table 1). Investigator #1 (laymen) was a student of the University of Applied Sciences FH Technikum Vienna, with no prior experience or skill in the field of musculoskeletal US diagnostics. Prior to this investigation, he received basic introduction in US diagnostic of the ATFL, consisting of 40 h hands-on tutorial training. Investigator #2 (medical student) was a fourth-year medical student of the Medical University of Vienna with basic US knowledge and training, imparted by medical school curriculum. Prior to this investigation, he had 1.5 years of professional experience in US musculoskeletal diagnostics. Investigator #3 (orthopedic specialist) was a specialist in sports orthopedics and orthopedic surgery with 20 years of experience in US diagnostics of the musculoskeletal apparatus. Investigator #4 (radiology specialist) was a specialist in radiology with a focus on musculoskeletal imaging, who had 15 years of experience in US imaging of the musculoskeletal apparatus.

**Table 1:** List of investigators tabulated by basic training, years of professional experience in US imaging, skill level and age

Investigator	Basic training	Years of professional experience in US imaging	Skill level	Age (yrs)
#1 Laymen	No	0	Laymen	23
#2 Medical student	Yes	1.5	Student	23
#3 Orthopedic specialist	Yes	20	Professional	55
#4 Radiology specialist	Yes	15	Expert	36

### Hardware2

All examinations were performed with a NextGen LOGIQ e Ultrasound console, operating a high-frequency linear array L8-18i-RS stick ultrasound probe by GE Healthcare (Company GE, 2014)Wauwatosa, Wisconsin, United States of America). The transducer possesses a footprint of 11.1 × 34.8 mm with a bandwidth of 6.7–18.0 MHz imaging frequency (General Electric Company, 2014). All examinations were performed at 18.0 MHz frequency.

### Transducer handling

**Fig. 1:**
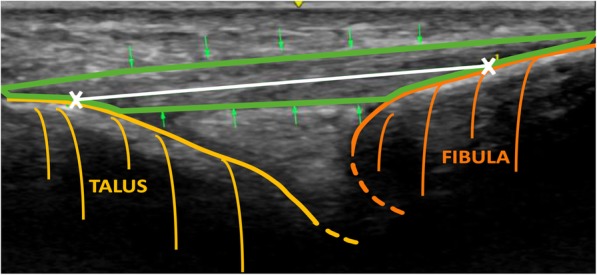
The illustration depicts the US image of the ATFL (green) in a linear extent between the lateral malleolus (orange) and lateral joint surface of the talus (yellow), corresponding to the bony attachment sites. White “X” and line represent the distance between the center of each insertion point

**Fig. 2:**
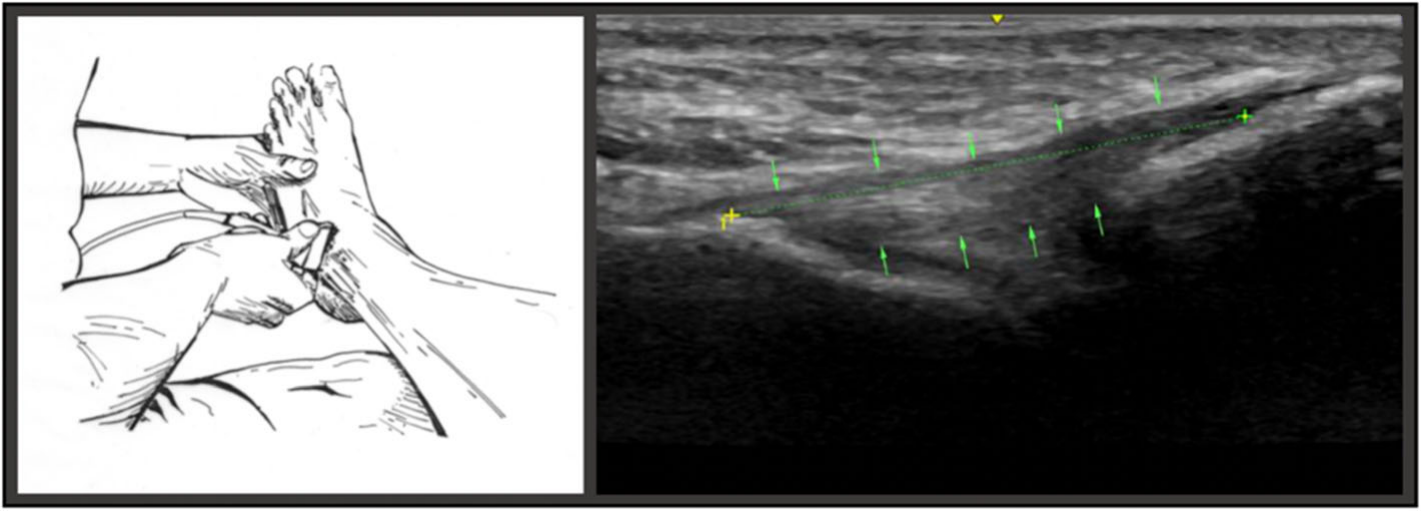
The illustration on the left portrays the examiner placing transducer on relaxed lateral ankle in plantarflexion of 10–20°. The image on the right depicts the measuring points of the ATFL in position A

**Fig. 3:**
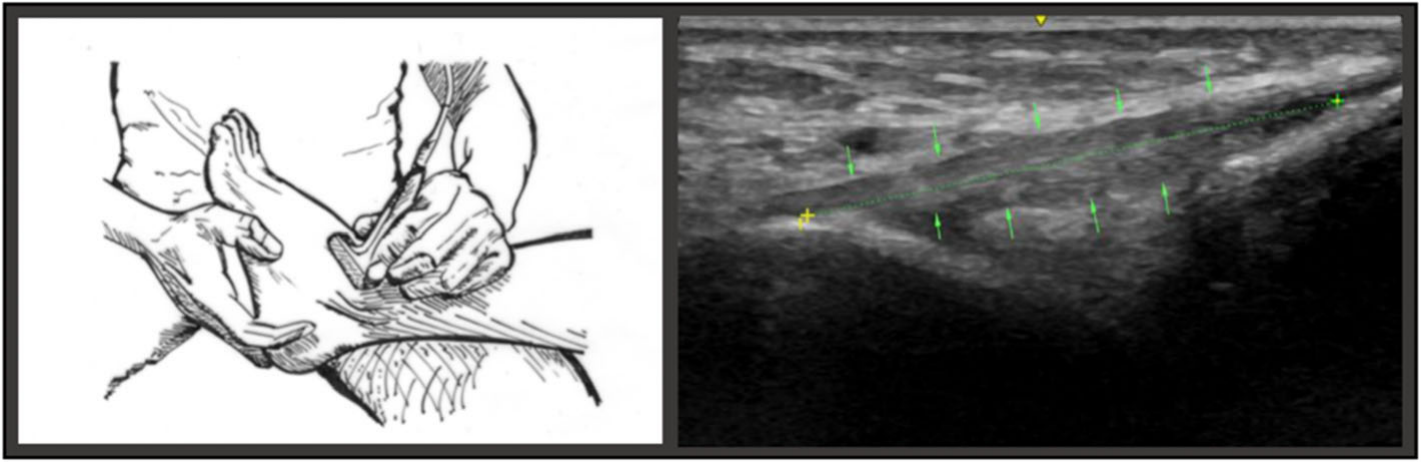
The illustration on the left portrays the examiner placing the transducer on the lateral ankle in with the foot in passive medial rotation. The image on the right depicts the measuring points of the ATFL in position B

**Fig. 4:**
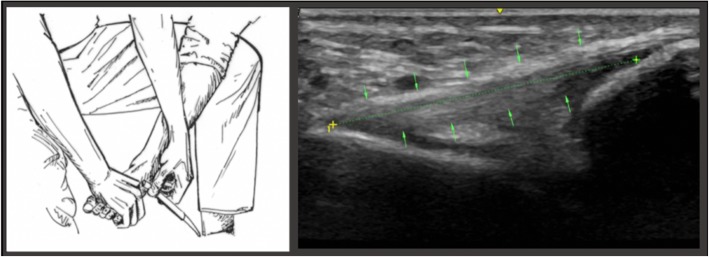
The illustration on the left portrays the examiner placing the transducer on the lateral ankle in maximal passive plantar flexion. The image on eh right depicts the measuring points of the ATFL in position C

To visualize the ATFL, the transducer was placed in the transversal plane, perpendicular to the subjects’ longitudinal axis. With the bony palpable malleolus lateralis as reference point, the transducer was mounted one cm proximal to the most distal palpable bony part of the fibula, projecting the left-aligned, convex, echogenic outline of the lateral malleolus on the imaging unit. Subsequently, employing this fixpoint, the transducer was slightly rotated radially along the longitudinal axis of the foot towards the talus, until its outline could be identified as an ascending echogenic line, opposing the fibula. This resulted in the sectional image, in which the lateral malleolus, talus and bridging ATFL were distinguished (Figs. 1, 2, 3, 4*).*

### Data collection

The length of the ATFL was appraised with the measure-utility of the LOGIQ e ultrasound console. To ensure reproducibility of the measurements, a linear extent between the lateral malleolus and the foremost point of the lateral joint surface of the talus, corresponding to the bony attachment sites of the ligament, were elected as described by Croy et al (Croy et al., 2012) After full visualization of ATFL width, the protocol was to elect the center of each ligament insertion point on the US cross-section (Fig. 1).

### Patient positioning

The measurements were conducted with the subjects in four distinctive positions. Each investigator autonomously visualized and measured the ATFL of all 16 subjects in each of the four widely used positions, three times per position to produce an average length per examiner per position. Between measurements, the subjects loosened their lower extremity to obviate probable distortions of range of motion by developing muscle tension.

In the first position (*position A*), the subject was seated on the examination table, with the calf of the designated lower extremity resting across the examiners knee and the ankle suspended in slight (10–20 degrees) plantarflexion. This position, also described by Cho, et al. 2015, was rendered the neutral resting position as baseline value for each subsequent measurement of the ATFL (Fig. 2).

To assume *position B*, the subject’s ankle was passively rotated medially by the investigator. The subject was seated on the examination table, with the calf of the designated lower extremity resting across the examiners knee and the ankle suspended. The measurement of the ATFL was conducted at maximal internal rotation stress of the talocrural joint (Fig.3) (Cho et al., 2016).

With the subject’s calf remaining on the examiners knee, *position C* was assumed through maximal plantarflexion of the ankle by the examiner (Fig. 4).

**Fig. 5:**
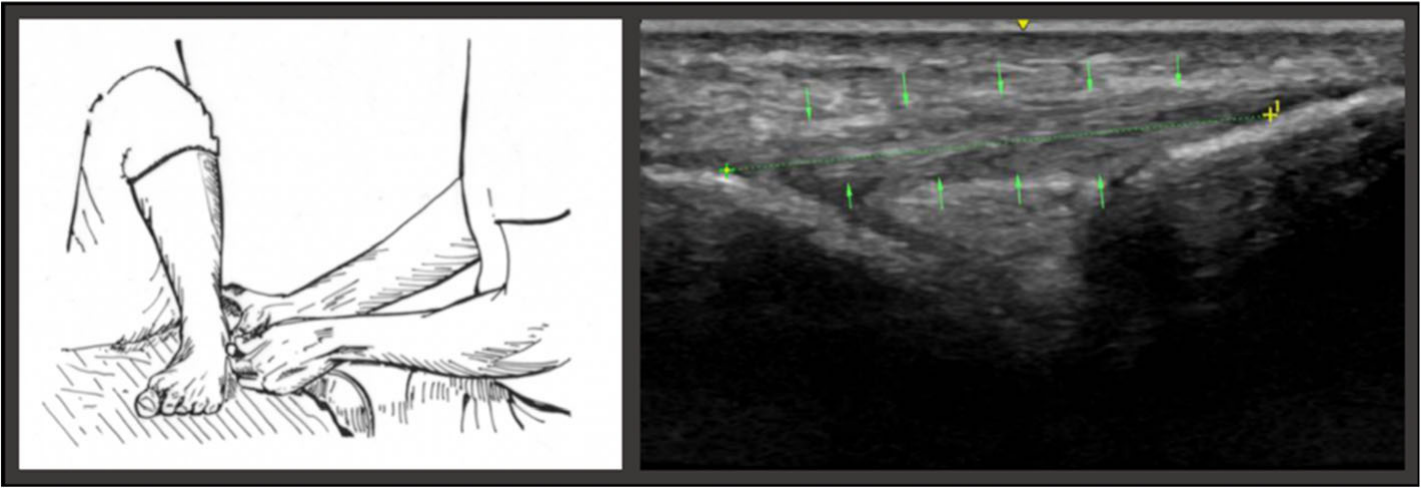
The illustration on the left depicts the examiner placing the transducer on the lateral ankle with the patient’s knee bent at 45° and the ankle maximally inverted. The image on the right shows the measuring points of the ATFL in position D

*Position B & C* can also be exhibited via widely used clinical examination protocols (DeLee & Miller, 2018; Campbell et al., 2017; Thompson & Miller, 2015). As demarcated standard patient positioning of the ATFL per European Society of Musculoskeletal Radiology (ESSR), *position D* required the patient sitting on the examination table with the knee bent 45 degrees and the sole of the foot placed flat on the examination table (Beggs et al., 2010; Lee & Yun, 2017). Next, the foot was placed in maximal inversion, so as to tense the lateral ligaments (Fig. 5).

### Data processing

To examine the reproducibility, interrater correlation of the investigators’ results for each position (A, B, C, D) was analyzed by means of Interclass Correlation Coefficient (ICC). As each measurement was carried out by deliberately selected investigators beforehand, the model with two-way mixed average measures was applied. For the statistical analysis, the software SPSS Statistics 25 by IBM (Armonk, New York, United States of America) and Microsoft Excel (Microsoft Corporation, Redmond, Washington, United States of America) were utilized. The absolute accordance of the measurement results was examined, whereby the ICC for each investigator per subject and position was determined, for establishing the grand average. ICC values at a standard confidence interval of 95% were evaluated via interrater-agreement measures by model of Cicchetti (Cicchetti, 1981).

To compare the mean change in length of the ATFL, the length of the ATFL at rest (position A) was first subtracted from the results of the stress tests (Position B, C and D). Subsequently, mean values and standard deviations of the length changes (Δl) were calculated for each position and then compared.

## Results

### Statistical analysis

For statistical computation and evaluation, we used the software SPSS 20 (IBM, USA). The correlation between the results of all four investigators was 0.333 for position A, 0.386 for position B, 0.320 for position C and 0.517 for position D. The highest correlation, independent from investigator, was 0.517 in position D.

Examining the ICC of investigators, regardless of position, the ICC was highest between investigator #3 and investigator #4 with 0.811, followed by 0.524 between investigator #2 and investigator #3. The lowest ICC was recorded between measurement results of investigator #1 and investigator #4, marking 0.299 (Table 2)

**Table 2:** Matrix indicating the ICC between each investigator regardless of the position

	Laymen	Medical student	Orthopedic specialist	Radiology specialist
Laymen (investigator #1)		0.390	0.355	0.299
Medical student (investigator #2)	0.390		0.524	0.390
Orthopedic specialist (investigator #3)	0.355	0.524		0.811
Radiology specialist (investigator #4)	0.299	0.390	0.811	

.

The length of the ATFL was 14.958 ± 2.145 mm in position A, 15.886 ± 1.994 mm in position B, 16.270 ± 1.858 mm in position C and 15.170 ± 1.781 mm in position D. The average length change was 0.928 ± 0.804 mm (6.656 ± 6.299%) in position B, 1.313 ± 1.266 mm (9.746 ± 9.484%) in position C and 0.213 ± 1.807 mm (2.604 ± 12.308%) in position D.

For investigators #1, #2, #3 & #4, the greatest average length variation (compared to resting position A) was recorded in position C and yielded 1.261 ± 0.806 mm (9.523 ± 7.829%), 1.644 ± 2.618 mm (13.300 ± 18.834%), 1.456 ± 0.906 mm (10.087 ± 6.311%) and 0.919 ± 0.732 mm (6.073 ± 4.964%) respectively.

Contrarily, the least average length variation was recorded in position D by all investigators, marking 0.575 ± 1.288 mm (5.128 ± 10.050%), 0.475 ± 2.673 mm (5.317 ± 18.713%), 0.131 ± 1.689 mm (1.447 ± 10.925%), − 0.331 ± 1.577 mm (− 1.476 ± 9.545%) respectively.

## Discussion

The most important finding of this study is that reliability of US measurement is dependent on examiner skill and experience. In the hand of an experienced examiner, US provides reliable and valuable results. The results of this study underline this assumption and the importance of teaching of ultrasonographic skills. Amid increasing evidence of the efficacy and accuracy of musculoskeletal apparatus US diagnostics, US diagnostic measures of injuries to the ATFL, experience incremental attention (Cai et al., 2017). Hua, et al. (2012) and Cheng, et al., (2014), investigated the quality of US examinations in the diagnosis of chronic ATFL injuries compared to arthroscopic findings and their surgery reports, respectively, with significant results in sensitivity, specificity and accuracy (Cheng et al., 2014). Lee, et al. (2012) and Cho, et al. (2016), compared the US stress-test in patients with chronic instability to the clinical stress-test, the radiographic stress-test, MRI and arthroscopy, defining US stress-testing as a viable additional diagnostic tool (Lee et al., 2014). Gün, et al. (2013), established that there is no significant difference in the diagnostic accuracy of diagnostic ultrasonography and MRI (Gun et al., 2013). Most recently, Lee (2017) and Cao (2018) conclude that point of care US, while as precise as MRI, may be cost saving in patient management and increasing quality of care, while it also is more accurate in chronic ankle instability (Lee & Yun, 2017; Cao et al., 2018; Radwan et al., 2016). Given this compelling evidence, one can safely argue that US imaging is a valuable diagnostic tool for physicians visualizing the lateral ankle ligaments of patients with supination trauma or chronic ankle instability. Our study is the first investigation to scrutinize the relationship of measurement results and examiner skill and experience. Furthermore, Furthermore, we are the first group to determine one position superior among 4 common patient positions.

Observing the ICC between the individual examiners, it appears that the reliability of the results of the laymen is poor at ICC of 0.390, 0.355 and 0.299, compared to the medical student, orthopedic specialist and radiology specialist, respectively. Without prior knowledge and skill in US imaging and merely basic introduction to hardware handling and patient positioning, the laymen failed to produce reliable measurements of the ATFL. The orthopedic specialist and radiology specialist presented an ICC of 0.811 (> 0.75), scoring the sole excellent correlation as per Cicchetti, 1981 (Cicchetti, 1981).

One can argue that their proximity of measurements reflects their reliability of diagnostic imaging by means of their long pedigree in US imaging. The ICC between the medical student and the laymen and radiology specialist indicated poor correlation. However, the medical student recorded sufficient correlation (0.524) with the orthopedic specialist. As measurements of the medical student showed higher correlation to the orthopedic specialist’s results than the measurements of the laymen, one can reason that the medical student’s basic skill and 1.5 years of professional experience with US imaging of the musculoskeletal apparatus, reflect the increasing reliability of his US measurements. Despite sufficient correlation with the orthopedic specialist, results of the medical student were barely commensurable with the results of the radiology specialist, charting at an ICC of 0.390. This may demonstrate the persistent gap in knowledge, experience and skill between the two investigators. Due to the sensitivity of the ultrasound system, approximate reliability presupposes abundant practice.

Exhibited by the results of this study, the medical student may have capitalized from basic US knowledge and skill imparted by his medical school curriculum, as well as 1.5 years of professional experience in US imaging, suggested in the sufficient reliability in marked contrast to the laymen. Per indication of increased diagnostic reliability with training and experience, the investment in training of ultrasonography evaluation in early medical career ought to be sustained and developed.

With regard to the examination position and ICC, a poor overall reliability (< 0.4, by Cicchetti, 1994) of the examination results was found in position A, B and C. One possible justification for this could be the 10–20 degrees of plantarflexion in the resting position (position A) specified by Cho, et al. (2016), providing a 10-degree margin of fluctuating levels of tension of the ATFL (Cho et al., 2016). Factors determining the accuracy of measurement in position B and C accumulate to investigator induced variation. In this position of medial rotation as well as plantarflexion, the examiner had to passively stress the talocrural joint. Therefore, variation of subjective joint loading via investigator force and grip cannot be discounted. Sufficient reliability (0.4–0-59, by Cicchetti 1994) could be observed in position D, the standard position described by the European Society of Musculoskeletal Radiology (ESSR). As the subject remained seated on the examination table with the determined leg flexed at 45 degrees in the knee and the foot placed flat on the table, the investigator’s task during this examination was reduced to accurate placement of the transducer with little degree of freedom to manipulate the joint angle. This slim margin for variation can be illustrated with the highest ICC (0.517) out of all four positions.

The highest elongation recorded among all positions, compared to the resting position, occurred in maximal plantarflexion. At an average length of 16.270 ± 1.858 and an absolute change in length of 1313 ± 1.266 mm, this position also exhibited the largest change in aforementioned values among each investigator. As briefly discussed above, measurement in this position arguably yielded elevated variability, born of various investigator grip and force, in addition to ankle soft tissue composition and individual ankle range of motion (Nigg et al., 1990).

One of the limitations of this study is the sample consisting of healthy pupils without injury history. Therefore, the significant results of this investigation are confined to the measurement reliability of US examination, influenced by investigator experience and skill, rather than diagnostic reliability. Therefore, considerations to substantiate clinical relevance of this investigation should include studies examining patients with acute and/or chronic ligament injury. As injury to the ATFL can present with varying injury patterns, increased sample size with analogous injury classifications could be planned. Nevertheless, as evidence proposes (Croy et al., 2013; Mizrahi et al., 2018; Bai et al., 2013) the length of the ATFL does in fact positively correlate with ligament injury, proving the US length measurement to be a practical diagnostic tool.

## Conclusion

Ultrasonographic imaging is a valuable diagnostic tool for physicians visualizing the lateral ankle ligaments as it reflects increasing reliability of measurements with examiner experience and skill. This suggests that investment in training of ultrasonography evaluation in early medical career ought to be sustained and developed. The patient positioning described by the European Society of Musculoskeletal Radiology seems to be least affected by examiner experience and skill, further deeming this position reliable for ultrasonographic examination.

## Data Availability

The datasets used and/or analysed during the current study are available from the corresponding author on reasonable request.

## Abbreviations

ATFL: Anterior talofibular ligament
ICC: Interclass correlation coefficient
MRI: Magnet resonance imaging
US: Ultrasound/ultrasonography

## References

[CR1] Bai L, Zhang WT, Huang W, Zhang XT, Jiang CQ, Li W (2013). Anatomical evaluation and clinical significance of lateral ankle ligament. Beijing Da Xue Xue Bao.

[CR2] Beggs I, Bianchi S, Bueno A, Cohen M, Court-Payen M, Andrew GFK et al (2010) Musculoskeletal ultrasound: technical guidelines. Insights Imaging (European Society of MusculoSkeletal Radiology):99–141

[CR3] Cai Y, Li S, Chen S, Hua Y, Shan J (2017). An ultrasound classification of anterior Talofibular ligament (ATFL) injury. The Open Orthopaedics J.

[CR4] Campbell WC, Canale ST, Beaty JH, Daugherty K, Jones L, Azar FM, Maxey S (2017). Campbells operative orthopaedics.

[CR5] Cao S, Wang C, Ma X, Wang X, Huang J, Zhang C (2018). Imaging diagnosis for chronic lateral ankle ligament injury: a systemic review with meta-analysis. J Orthop Surg Res.

[CR6] Cheng Y, Cai Y, Wang Y (2014). Value of ultrasonography for detecting chronic injury of the lateral ligaments of the ankle joint compared with ultrasonography findings. Br J Radiol.

[CR7] Cho JH, Lee DH, Song HK, Bang JY, Lee KT, Park YU (2016). Value of stress ultrasound for the diagnosis of chronic ankle instability compared to manual anterior drawer test, stress radiography, magnetic resonance imaging, and arthroscopy. Knee Surg Sports Traumatol Arthrosc.

[CR8] Cicchetti DS (1981) S. Developing criteria for establishing interrater reliability of specific items: applications to assessment of adaptive behavior. Am J Ment Defic:127–137 7315877

[CR9] Company GE. New LOGIQ e Ultrasound System Transducer Guide. 2014

[CR10] Croy T, Saliba S, Saliba E, Anderson MW, Hertel J (2013). Talofibular interval changes after acute ankle sprain: a stress ultrasonography study of ankle laxity. J Sport Rehabil.

[CR11] Croy T, Saliba SA, Saliba E, Anderson MW, Hertel J (2012). Differences in lateral ankle laxity measured via stress ultrasonography in individuals with chronic ankle instability, ankle sprain copers, and healthy individuals. J Orthop Sports Phys Ther.

[CR12] Delahunt E, Bleakley CM, Bossard DS (2018). Clinical assessment of acute lateral ankle sprain injuries (ROAST): 2019 consensus statement and recommendations of the international ankle consortium. Br J Sports Med.

[CR13] DeLee JDD, Miller MD (2018). Orthopaedic sports medicine: principles and practice.

[CR14] Friedrich JM, Heuchemer T, Schumacher KA, Bargon G (1990). The use of sonography in the diagnosis of fresh talofibular ligamentous lesions. RoFo.

[CR15] Gun C, Unluer EE, Vandenberk N, Karagoz A, Senturk GO, Oyar O (2013). Bedside ultrasonography by emergency physicians for anterior talofibular ligament injury. J Emergencies, Trauma, Shock.

[CR16] Kerkhoffs GM, Handoll HH, de Bie R, Rowe BH, Struijs PA (2002) Surgical versus conservative treatment for acute injuries of the lateral ligament complex of the ankle in adults. Cochrane Database Syst Rev (3):Cd000380 10.1002/14651858.CD000380 12137612

[CR17] Lee KT, Park YU, Jegal H, Park JW, Choi JP, Kim JS (2014). New method of diagnosis for chronic ankle instability: comparison of manual anterior drawer test, stress radiography and stress ultrasound. Knee Surg Sports Traumatol Arthrosc.

[CR18] Lee SH, Yun SJ (2017). The feasibility of point-of-care ankle ultrasound examination in patients with recurrent ankle sprain and chronic ankle instability: comparison with magnetic resonance imaging. Injury..

[CR19] Mizrahi DJ, Nazarian LN, Parker L (2018) Evaluation of the anterior Talofibular ligament via stress sonography in asymptomatic and symptomatic populations. J Ultrasound Med 10.1002/jum.14542 29363788

[CR20] Nigg BM, Skarvan G, Frank CB, Yeadon MR (1990). Elongation and forces of ankle ligaments in a physiological range of motion. Foot & ankle.

[CR21] Radwan A, Bakowski J, Dew S, Greenwald B, Hyde E, Webber N (2016). Effectiveness of ultrasonography in diagnosing chronic lateral ankle instability:a systematic review. Int J Sports Phys Ther.

[CR22] Roos KG, Kerr ZY, Mauntel TC, Djoko A, Dompier TP, Wikstrom EA (2017). The epidemiology of lateral ligament complex ankle sprains in National Collegiate Athletic Association Sports. Am J Sports Med.

[CR23] Thompson SR, Miller MD (2015) Millers review of Orthopaedics. Elsevier

[CR24] Walther M, Kriegelstein S, Altenberger S, Volkering C, Roser A, Wolfel R (2013). Unfallchirurg.

